# Miro1-mediated mitochondrial positioning supports subcellular redox status

**DOI:** 10.1016/j.redox.2020.101818

**Published:** 2020-11-29

**Authors:** Haya Alshaabi, Nathaniel Shannon, Randi Gravelle, Stephanie Milczarek, Terri Messier, Brian Cunniff

**Affiliations:** Department of Pathology and Laboratory Medicine, University of Vermont Cancer Center, Larner College of Medicine, Burlington, VT 05405, USA

**Keywords:** Mitochondrial trafficking, Reactive oxygen species, Hydrogen peroxide, Miro1, Cell migration

## Abstract

Mitochondria are strategically trafficked throughout the cell by the action of microtubule motors, the actin cytoskeleton and adapter proteins. The intracellular positioning of mitochondria supports subcellular levels of ATP, Ca^2+^ and reactive oxygen species (ROS, i.e. hydrogen peroxide, H_2_O_2_). Previous work from our group showed that deletion of the mitochondrial adapter protein Miro1 leads to perinuclear clustering of mitochondria, leaving the cell periphery devoid of mitochondria which compromises peripheral energy status. Herein, we report that deletion of Miro1 significantly restricts subcellular H_2_O_2_ levels to the perinuclear space which directly affects intracellular responses to elevated mitochondrial ROS. Using the genetically encoded H_2_O_2_-responsive fluorescent biosensor HyPer7, we show that the highest levels of subcellular H_2_O_2_ map to sites of increased mitochondrial density. Deletion of Miro1 or disruption of microtubule dynamics with Taxol significantly reduces peripheral H_2_O_2_ levels. Following inhibition of mitochondrial complex 1 with rotenone we observe elevated spikes of H_2_O_2_ in the cell periphery and complementary oxidation of mitochondrial peroxiredoxin 3 (PRX3) and cytosolic peroxiredoxin 2 (PRX2). Conversely, in cells lacking Miro1, rotenone did not increase peripheral H_2_O_2_ or PRX2 oxidation but rather lead to increased nuclear H_2_O_2_ and an elevated DNA-damage response. Lastly, local levels of HyPer7 oxidation correlate with the size and abundance of focal adhesions (FAs) in MEFs and cells lacking Miro1 have significantly smaller focal adhesions and reduced phosphorylation levels of vinculin and p130Cas compared to Miro1^+/+^ MEFs. Together, we present evidence that the intracellular distribution of mitochondria influences subcellular H_2_O_2_ levels and local cellular responses dependent on mitochondrial ROS.

## Graphical abstract

**Figure:**
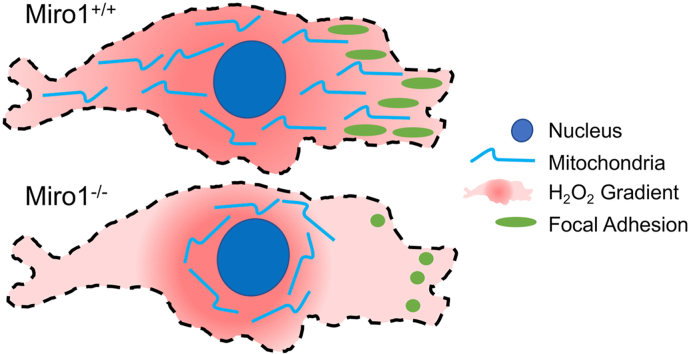

## 1 Introduction

Mitochondria are dynamic organelles that vary in size, shape and location depending on cell type, energy status and metabolic demand for mitochondrial metabolites [1]. The shape and distribution of the mitochondrial network is partly controlled by subcellular cues that respond to changes in energy needs [2,3], Ca^2+^ transients [[4], [5], [6]], redox status [7] and post-translational modifications in regulatory proteins [8]. Interactions between mitochondria and other cell structures (actin, ER, endosomes) further influences mitochondrial structure and location [9]. Mitochondria redistribute throughout the cell cytoplasm by the action of the microtubule-associated motors, kinesin and dynein [[10], [11], [12]]. Mitochondria coupling to kinesin and dynein is mediated by a protein complex consisting of TRAK1/2 and the mitochondrial Rho GTPases Miro1 and Miro2 (Rhot 1/2) [[13], [14], [15], [16], [17], [18]]. Miro1 is a C-terminal anchored mitochondrial outer membrane protein required for subcellular mitochondrial distribution, and thereby supports neuronal health [6,10,19], immune responses [[20], [21], [22]] and cell migration [2,[23], [24], [25]]. It is important to note that Miro1 is required for normal mitochondrial cristae architecture [26] but mitochondrial bioenergetics of Miro1^−/−^ mouse embryonic fibroblasts (MEFs) are indistinguishable from Miro1^+/+^ MEFs [24,26,27]. We have recently shown that deletion of Miro1, the primary protein required for redistribution of mitochondrial networks to the cell periphery in MEFs [28], restricts mitochondria to the perinuclear space and thereby alters subcellular energy levels [24]. Although Miro1 and Miro2 share ~60% sequence identity, deletion of Miro2 does not alter subcellular mitochondrial position in MEFs [28]. Together, the subcellular distribution of mitochondria is critical for localized production of mitochondrial outputs and localized calcium buffering in numerous cells and tissues [29].

ROS are produced from several cellular sources including enzyme specific production of ROS by NADPH oxidases (NOXs) and from mitochondria during oxidative phosphorylation [30,31]. Approximately 0.15%–2% of the total electron flow through the mitochondrial electron transport chain (ETC) supports generation of mitochondrial ROS (mROS) [32], accounting for ~30% of cell derived extracellular H_2_O_2_ [33]. ETC complexes are arranged spatially in a hierarchy of redox potential, although the transfer of electrons between complexes is not confined to a closed system, as thermodynamically all ETC complexes can effectively reduce molecular oxygen [34]. This caveat allows electrons derived primarily from complexes I and III to react readily with molecular oxygen to form superoxide (O_2_^.-^) in the mitochondrial matrix and the inner membrane space, respectively [35]. Superoxide is rapidly converted to hydrogen peroxide (H_2_O_2_) by superoxide dismutases (SODs).

H_2_O_2_, which is diffusible through cellular membranes, but also can be transported through membrane channels, plays a central role in redox-dependent signaling cascades [36]. ROS, like H_2_O_2_, regulate cellular physiology through direct oxidation of cysteine residues in target proteins, or via inactivation of resident scavenger/chaperone proteins [37]. Specific, structurally distinct and solvent accessible cysteine residues are targets for oxidation by H_2_O_2_, and these modifications result in structural and functional changes in target proteins [38]. Similar to protein phosphorylation/dephosphorylation cascades, the reversible oxidation of specific cysteine residues modulates signaling pathways that govern all facets of cell physiology [36]. Physiochemical characteristics of oxidized cysteine residues in target proteins underlie the specificity and hierarchy of responses in redox signaling.

Similar to ATP, ROS are rapidly consumed at sites proximal to their source, largely due to the abundance of antioxidant enzymes present in the cell [38,39]. An additional level of regulation is achieved through the compartmentalization of oxidant and antioxidant systems, allowing cells to utilize redox-dependent systems for physiological signaling and damage responses while protecting redox-sensitive cell compartments [40,41]. For example, gradients of H_2_O_2_ have been observed in zebrafish tissues. Tail fin amputation leads to a 150–300 μm wide H_2_O_2_ gradient (further refined to 30 μm with updated approaches [39]) extending from the wound margin into the tissue [42]. This NADPH oxidase associated H_2_O_2_ gradient acts as a chemoattractant for inflammatory cell recruitment. H_2_O_2_ levels are elevated in migrating tumor cells compared to stationary cells, with elevated H_2_O_2_ in cell protrusions versus the cell body [43]. Oxidation of actin filaments specifically in cell protrusions has recently been described using the ratiometric H_2_O_2_ biosensor HyPer7 fused to the actin binding peptide LifeAct. Using this probe, protrusions with elevated H_2_O_2_ levels were more stable compared to protrusions with lower H_2_O_2_ levels [40]. Mitochondrial fragmentation following plasma membrane damage increases mROS levels that lead to RhoA mediated F-actin polymerization at the injury site [44]. In experiments using *C.elegans* epithelial cells, the process of increased mROS at wound sites was shown to be Miro1 dependent [45]. Additional evidence for mitochondrial control over subcellular ROS levels was shown in migrating tumor cells. Downregulation of SIRT3 (which promotes energy homeostasis) specifically in the leading-edge of migrating cells increased local mROS levels that promote tumor cell metastasis through modulation of focal adhesion kinase (FAK) signaling [46]. The local production and metabolism of H_2_O_2_ is therefore necessary for the correct spatial and temporal control of redox-responsive signaling events [41].

In this study, we evaluated the relationship between Miro1-mediated mitochondrial trafficking and subcellular H_2_O_2_ levels using the genetically encoded H_2_O_2_-responsive fluorescent biosensor HyPer7 [40]. Our results show that perinuclear restriction of mitochondria in Miro1 null (Miro1^−/−^) MEFs significantly reduces peripheral H_2_O_2_ levels. In response to increased mROS following complex 1 inhibition by rotenone, H_2_O_2_ levels are increased in the periphery of cells with intact Miro1. Conversely, rotenone did not increase peripheral H_2_O_2_ levels in Miro1^−/−^ MEFs but rather increased nuclear H_2_O_2_ levels and elevated DNA-damage responses. We further show phosphorylation of key proteins required for focal adhesion (FA) dynamics are decreased in Miro1^−/−^ MEFs and that subcellular areas of increased H_2_O_2_ levels correlates with FA size and number Together, our results provide evidence that the intracellular positioning of functional mitochondria regulates subcellular H_2_O_2_ levels in the leading edge periphery.

## 2 Results

### 2.1 Miro1 expression dictates peripheral mitochondrial density

**Fig. 1:**
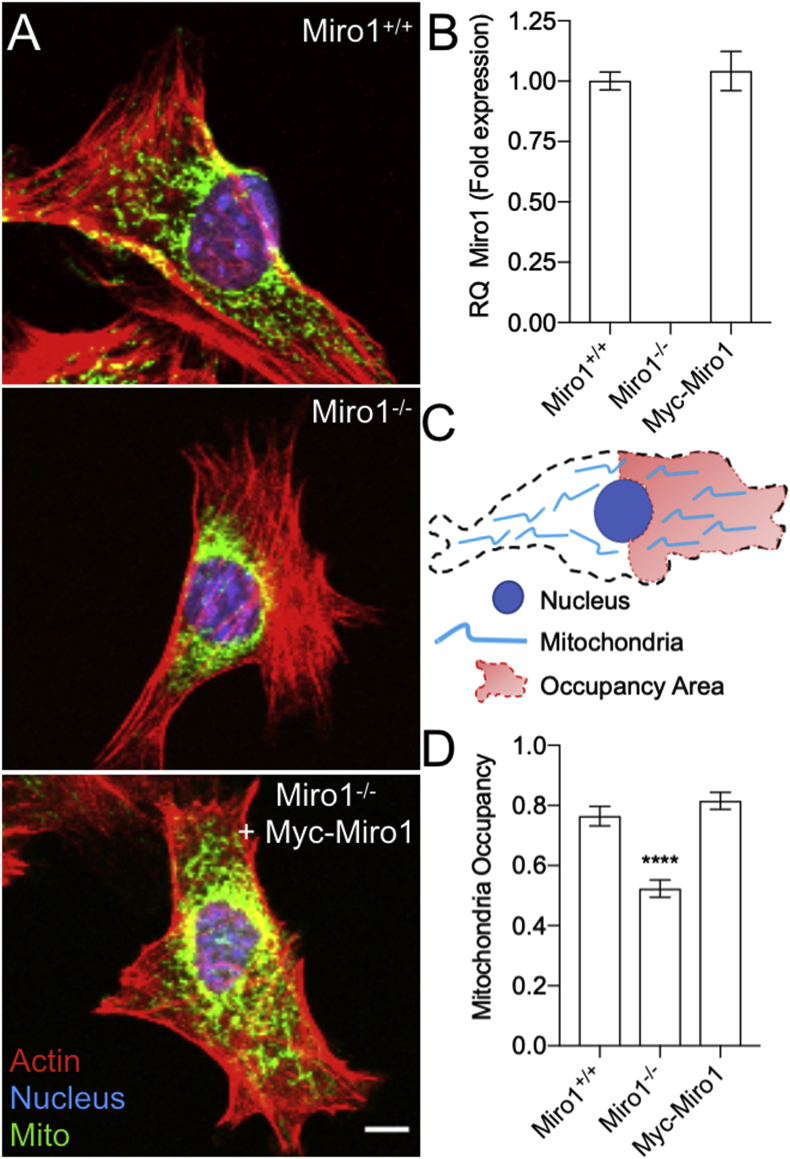
Mitochondria are restricted around the nucleus in cells lacking Miro1 (A) (Top) Mitochondria occupy the perinuclear and peripheral space in mouse embryonic fibroblasts (MEFs). (Middle) Mitochondria are restricted around the nucleus in MEFs devoid of Miro1 expression (Miro1^−/−^). (Bottom) Re-expression of Myc-Miro1 in Miro1^−/−^ cells rescues mitochondrial distribution to the cell periphery. (B) Miro1 mRNA expression levels determined by RT-qPCR with primers spanning Miro1 exons 2–4. (C) Schematic to describe cell area used for quantification of mitochondrial occupancy in the leading edge (LE) (Mitochondrial Area ÷ LE Area). (D) Quantification of mitochondrial occupancy in the leading edge of indicated MEFs (****p < 0.0001; one-way ANOVA with Tukey's posttest). Error bars show ± SE.

Miro1 deletion in MEFs leads to the perinuclear clustering of mitochondria, leaving the cell periphery devoid of mitochondrial populations [24,27,28] (Fig. 1). Importantly, mitochondria from Miro1^−/−^ MEFs retain mitochondrial bioenergetic properties comparable to cells with intact Miro1 (Miro1^+/+^) [24,26,27], and generate comparable levels of H_2_O_2_ as determined by measurement of extracellular H_2_O_2_ levels using Amplex Red (Supplementary Fig. 1), providing a system that allows for the investigation of effects solely related to the position of functional mitochondria. To rescue defects in the subcellular positioning of mitochondria, we generated a stable cell line by transfecting Miro1^−/−^ MEFs with Myc-Miro1 plasmid DNA (Fig. 1, referred to as Myc-Miro1 MEFs herein). Myc-Miro1 MEFs express Miro1 to comparable levels as Miro1^+/+^ MEFs (Fig. 1). We next confirmed the distribution patterns of mitochondria in the cell periphery in our 3 cell lines. The density of mitochondria in the leading-edge periphery of Miro1^−/−^ MEFs is significantly reduced compared to Miro1^+/+^ and Myc-Miro1 MEFs (Fig. 1). Therefore, we utilized these cell lines to investigate subcellular H_2_O_2_ levels.

### 2.2 Visualization of subcellular H_2_O_2_ levels using HyPer7

**Fig. 2:**
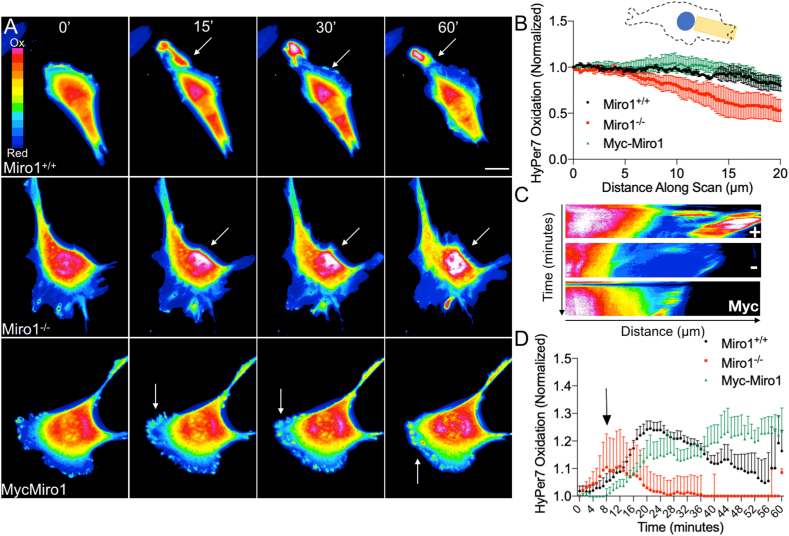
Visualization of subcellular H_2_O_2_ levels using HyPer7. (A) HyPer7 oxidation status at baseline (0 min) and following 1 μM Rot exposure at given timepoints (15–60 min). Arrows indicate areas of increased oxidation following Rot (scale bar = 20 μm). (B) Schematic of cell area used to evaluate HyPer7 oxidation in the leading edge. Distribution of HyPer7 oxidation from perinuclear space (0 μm) to peripheral leading edge (10–20 μm). Values normalized to highest HyPer7 ratio for each cell (n = 5–10 cells) (C) Kymographs of HyPer7 oxidation over time (0–60 min) following addition of 1 μM Rot. (D) Quantification of average HyPer7 oxidation in peripheral space over time following Rot exposure. Average HyPer7 oxidation in peripheral space normalized to initial value for each cell line (n = 5–10 cells). Arrow indicates timing of Rot addition. Error bars show ± SE.

To evaluate subcellular H_2_O_2_ levels in our cell lines we utilized the recently described genetically encoded H_2_O_2_-responsive biosensor HyPer7. HyPer7 is a pH stable genetically encoded biosensor with specificity for H_2_O_2_ and ultrafast reaction kinetics [40]. HyPer7 has two excitation peaks (400-nm and 499-nm) with maximal emission at 516-nm. The emission intensity after excitation at 400-nm and 499-nm changes based on the oxidation state of the probe. Emission increases following excitation at 499-nm when the probe is oxidized by H_2_O_2_ and therefore a higher numerical ratio indicates an increase in the local oxidation state of the probe and therefore an increase in local H_2_O_2_ levels. MEFs transfected with HyPer7 were visualized by live-cell fluorescent microscopy (Fig. 2). HyPer7 is expressed in both the cytosol and nuclear compartments [40] (Fig. 2). The highest HyPer7 signal was observed at perinuclear positions in Miro1^+/+^, Miro1^−/−^ and Myc-Miro1 MEFs (Fig. 2A). We quantified the change in H_2_O_2_ levels from the perinuclear space to the cell periphery in all cell lines and show that HyPer7 oxidation levels reproducibly decrease more rapidly at peripheral sites in Miro1^−/−^ MEFs as compared to Miro1^+/+^ or Myc-Miro1 MEFs (Fig. 2A, and B), thereby mapping basal H_2_O_2_ levels to the sites of observed mitochondrial populations in these cells (Fig. 1). To confirm that HyPer7 is responding to H_2_O_2_ uniformly in our cell lines we incubated Miro1^+/+^ and Miro1^−/−^ MEFs with the H_2_O_2_ generating enzyme Glucose Oxidase (GOx) and monitored HyPer7 response. HyPer7 is rapidly oxidized throughout the entire volume of the cell in both Miro1^+/+^ and Miro1^−/−^ MEFs following addition of 10 mU/mL GOx (Supplementary Fig. 1). Together, these results are consistent with earlier observations that showed peripheral ATP levels are lower in Miro1^−/−^ MEFs as compared to Miro1^+/+^ MEFs [24], providing visual evidence that the positioning of mitochondria by Miro1 influences subcellular ATP and H_2_O_2_ levels.

### 2.3 Subcellular response to mitochondrial ROS in relationship to mitochondrial distributions

We next increased mROS levels in MEFs using the mitochondrial complex 1 inhibitor rotenone (Rot) and evaluated the subcellular response of HyPer7. Rot inhibits electron transport by complex 1 of the electron transport chain (ETC) leading to elevated mROS production [47]. Miro1^+/+^ and Myc-Miro1 expressing MEFs showed a detectable increase in the perinuclear/cytoplasmic oxidation status of HyPer7 at 15-, 30- and 60-min post Rot exposure (Fig. 2A, C &D and Supplemental Movie 1). Oxidation spikes were observed in the peripheral cortical cytoskeleton at leading edge protrusions and actin ruffles following Rot treatment in Miro1^+/+^ and Myc-Miro1 MEFs (Fig. 2A). In contrast, oxidation events in the periphery of Miro1^−/−^ MEFs were significantly less abundant or non-existent at all time points following Rot exposure (Fig. 2A). Elevated HyPer7 oxidation in the cell periphery was sustained throughout the 60-min experiment in Miro1^+/+^ and Myc-Miro1 MEFs while no sustained HyPer7 oxidation was observed in Miro1^−/−^ MEFs (Fig. 2A, C, D and Supplemental Movie 1). Of interest, the oxidation state of the nuclear area in Miro1^−/−^ MEFs increased following Rot exposure which did not occur in Miro1^+/+^ MEFs and was less pronounced in Myc-Miro1 MEFs (Supplemental Fig. 2). Increased nuclear oxidation state at baseline and following Rot exposure in Miro1^−/−^ MEFs was also detectable by quantifying the intensity of nuclear H2AX phosphorylation in MEFs, a readout of DNA damage in response to oxidative stress [48]. H2AX phosphorylation is increased in Miro1^−/−^ MEFs at baseline compared to Miro1^+/+^ and Myc-Miro1 MEFs and is further elevated following Rot exposure (Supplemental Fig. 2).

**Fig. 3:**
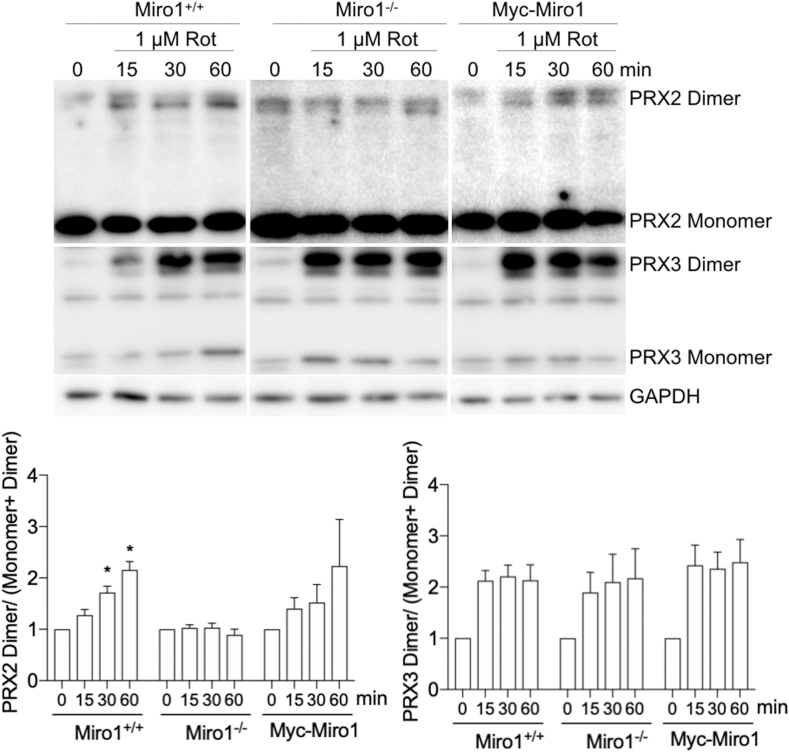
Rotenone increases mitochondrial and non-mitochondrial PRX oxidation. Non-reducing western blots of cytoplasmic PRX2 and mitochondrial PRX3 monomers and oxidized dimers in Miro1^+/+^, Miro1^−/−^ and Myc-Miro1 MEFs treated with 1 μM rotenone (Rot) for indicated periods of time. Quantification (Densitometry of Western blot bands) of cytosolic PRX2 and mitochondrial PRX3 oxidation state (oxidized dimer/monomer + dimer) from non-reducing western blots (n = 3 replicates, *p < 0.05; one-way ANOVA with Tukey's posttest). Error bars show ± SE.

To complement our imaging approaches we also evaluated the oxidation of mitochondrial peroxiredoxin 3 (PRX3) and cytosolic peroxiredoxin 2 (PRX2) at baseline and following Rot exposure using non-reducing western blotting approaches [49]. In response to increasing ROS levels and during catalysis of H_2_O_2_, a disulfide bond is formed between two PRX monomers. Oxidation of PRX monomers leads to the formation of disulfide-bonded dimers that can be visualized and quantified using non-reducing SDS-PAGE gels. This methodology provides a biochemical readout of local PRX oxidation state [49]. The oxidation of PRX3 to disulfide-bonded dimers increases to equal levels in all 3 cell lines following Rot exposure, indicating the response to Rot and induction of mROS was comparable between cell lines (Fig. 3). In line with our imaging data, the oxidation of cytosolic PRX2 is increased in Miro1^+/+^ and Myc-Miro1 MEFs following Rot exposure while no PRX2 oxidation is observed in Miro1^−/−^ MEFs (Fig. 3).These data provide quantitative fluorescent imaging and biochemical evidence that perinuclear restriction of mitochondrial populations in Miro1^−/−^ MEFs alters cellular response to increased mitochondrial derived H_2_O_2_.

### 2.4 Subcellular H_2_O_2_ levels at sites of mitochondrial occupancy

**Fig. 4:**
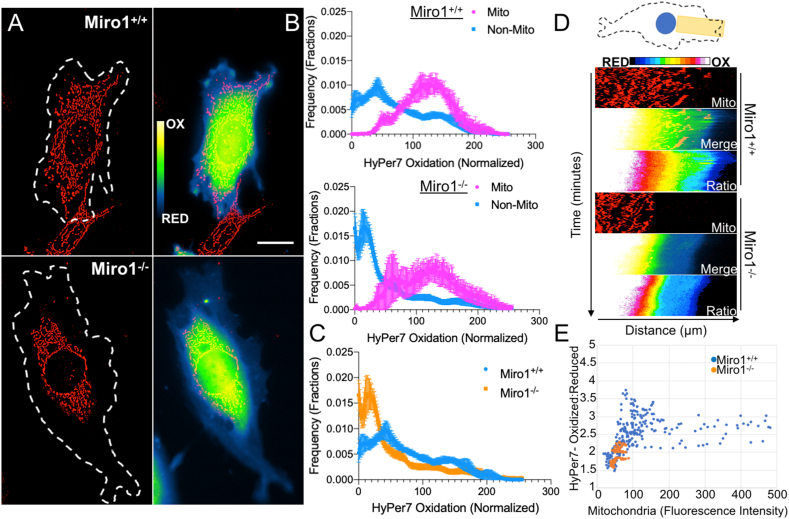
Subcellular H_2_O_2_ maps to sites of mitochondrial density (A) Visualization of HyPer7 oxidation and mitochondria labeled with MitoTracker Deep Red in Miro1^+/+^ and Miro1^−/−^ MEFs. Cell outline to visualize subcellular distribution of mitochondria. Scale bar = 20 μm. (B) Histograms of mitochondrial (Mito) and non-mitochondrial (Non-Mito) associated HyPer7 oxidation in Miro1^+/+^ (Top) and Miro1^−/−^ (Bottom) MEFs. 0 = low Hyper7 oxidation events, 300 = high HyPer7 oxidation events (n = 6 cells/group). (C) Distribution of Non-Mito HyPer7 oxidation levels in Miro1^+/+^ and Miro1^−/−^ MEFs (n = 6 cells/group). (D) Schematic of area used to project leading edge kymographs below. Mitochondria, Mitochondria + HyPer7 oxidation and HyPer7 oxidation over time (0–60 min) in Miro1^+/+^ and Miro1^−/−^ MEFs. (E) Correlation between mitochondrial fluorescence and HyPer7 oxidation status in the leading edge of Miro1^+/+^ and Miro1^−/−^ MEFs. Error bars show ± SE. (For interpretation of the references to colour in this figure legend, the reader is referred to the Web version of this article.)

To specifically investigate the connection of subcellular H_2_O_2_ levels with mitochondrial position we imaged HyPer7 expressing cells co-stained with the fluorescent mitochondrial dye MitoTracker Deep Red (Fig. 4A). We evaluated HyPer7 oxidation state at areas associated with mitochondria and at areas not associated with mitochondria in Miro1^+/+^ and Miro1^−/−^ MEFs (See Supplemental Fig. 3 for methodology). Histograms of the distribution of HyPer7 oxidation state were normalized to the lowest signal in each cell and normalized values from each genotype were combined for analysis. Levels of HyPer7 oxidation at sites directly associated with mitochondria (Mito) were similar between Miro1^+/+^ and Miro1^−/−^ MEFs (Fig. 4A and B). Both genotypes showed a small but distinct population of mitochondria with reduced HyPer7 oxidation (at ~60 units on X-axis) (Fig. 4B), highlighting the heterogeneity H_2_O_2_ levels associated with individual mitochondria. The levels of HyPer7 oxidation at areas not associated with mitochondria (Non-Mito) were lower compared to mitochondrial associated HyPer7 oxidation status in both cell lines (Fig. 4B). However of importance, the distribution of HyPer7 oxidation levels in Miro1^+/+^ MEFs was bimodal, with some elevated HyPer7 oxidation events occurring at sites not directly associated with mitochondria (Fig. 4B and C) while no elevated HyPer7 oxidation events occurred at sites not associated with mitochondria in Miro1^−/−^ MEFs (Fig. 4B and C). We next evaluated HyPer7 oxidation in relationship to mitochondrial density in Miro1^+/+^ and Miro1^−/−^ MEFs over time (Fig. 4D and E and Supplemental Movie 2). Kymographs of mitochondrial dynamics and HyPer7 oxidation status over a 60-min time scale were generated to visualize and quantify mitochondrial density and HyPer7 oxidation (Fig. 4D). Mitochondria actively traffic into the leading edge of Miro1^+/+^ MEFs and this corresponds with sustained HyPer7 oxidation in this compartment. In Miro1^−/−^ MEFs, mitochondria remain restricted around the perinuclear region corresponding with constricted HyPer7 oxidation (Fig. 4D). We next plotted HyPer7 oxidation status in relationship to mitochondrial density over time in leading edge membranes (Fig. 4E). Data points from Miro1^−/−^ MEFs are clustered to low HyPer7 oxidation and low mitochondrial density, as the leading edge of Miro1^−/−^ MEFs are devoid of mitochondria. A linear relationship is observed in Miro1^+/+^ MEFs where sites of increasing mitochondrial density show higher levels of HyPer7 oxidation (Fig. 4E). Of note, the brightest mitochondria do not always indicate elevated HyPer7 oxidation, this may be attributed to differences in mitochondrial membrane potential (increased MitoTracker Deep Red accumulation), mitochondrial shape changes, heterogeneity in H_2_O_2_ levels associated with individual mitochondria or other factors not investigated.

### 2.5 Microtubule disruption influences mitochondrial distribution and subcellular HyPer7 oxidation

**Fig. 5:**
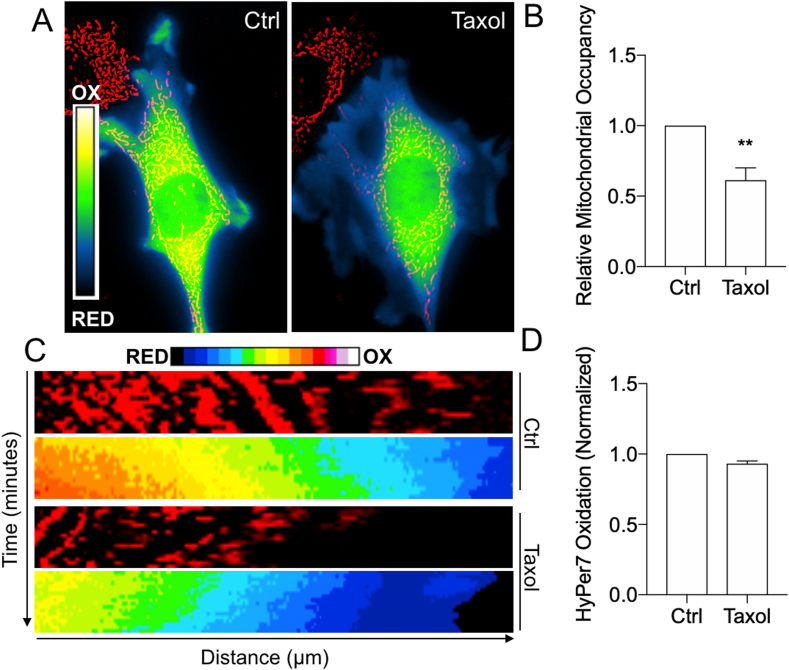
Taxol decreases peripheral H_2_O_2_ levels. (A) Control and Taxol treated Miro1^+/+^ MEFs expressing HyPer7 and stained with MitoTracker Deep Red. (B) Relative mitochondrial occupancy in the peripheral leading edge of Miro1^+/+^ MEFs treated with Taxol (**p < 0.01, Student's *t*-test). (C) Kymographs of leading-edge membranes from control and Taxol treated cells over 60 min. (D) Quantification of total HyPer7 oxidation in Ctrl and Taxol treated cells. Error bars show ± SE. (For interpretation of the references to colour in this figure legend, the reader is referred to the Web version of this article.)

Because Miro1 may be important for other mitochondrial or cellular processes [26,50], we utilized compounds that disrupt the microtubule cytoskeleton which is required for mitochondrial trafficking [2,28]. In our previous work, treatment of cells with Taxol or nocodazole (Noco) significantly reduced mitochondrial flux into leading edge membranes [2]. Here, we blocked mitochondrial trafficking in Miro1^+/+^ MEFs using Taxol [2] and evaluated leading edge HyPer7 oxidation (Fig. 5). Taxol significantly reduced mitochondrial occupancy in the cell periphery within 15 min with concomitant loss of HyPer7 oxidation in the peripheral cytoskeleton to levels comparable to Miro1^−/−^ MEFs (Fig. 5A and B). Taxol had no effect on the subcellular distribution of HyPer7 oxidation in Miro1^−/−^ MEFs, with both mitochondria and H_2_O_2_ levels staying perinuclear restricted (Supplemental Fig. 4). Mitochondrial restriction and reduced HyPer7 oxidation in the cell periphery was sustained for the entirety of the treatment (60 min) (Fig. 5C). Total levels of HyPer7 oxidation throughout the cell were not significantly altered by Taxol indicating Taxol did not directly affect the production of H_2_O_2_ from mitochondria or other sources (Fig. 5D). These data support our findings in Miro1^−/−^ MEFs that subcellular positioning of mitochondria dictates peripheral H_2_O_2_ levels.

**Fig. 6:**
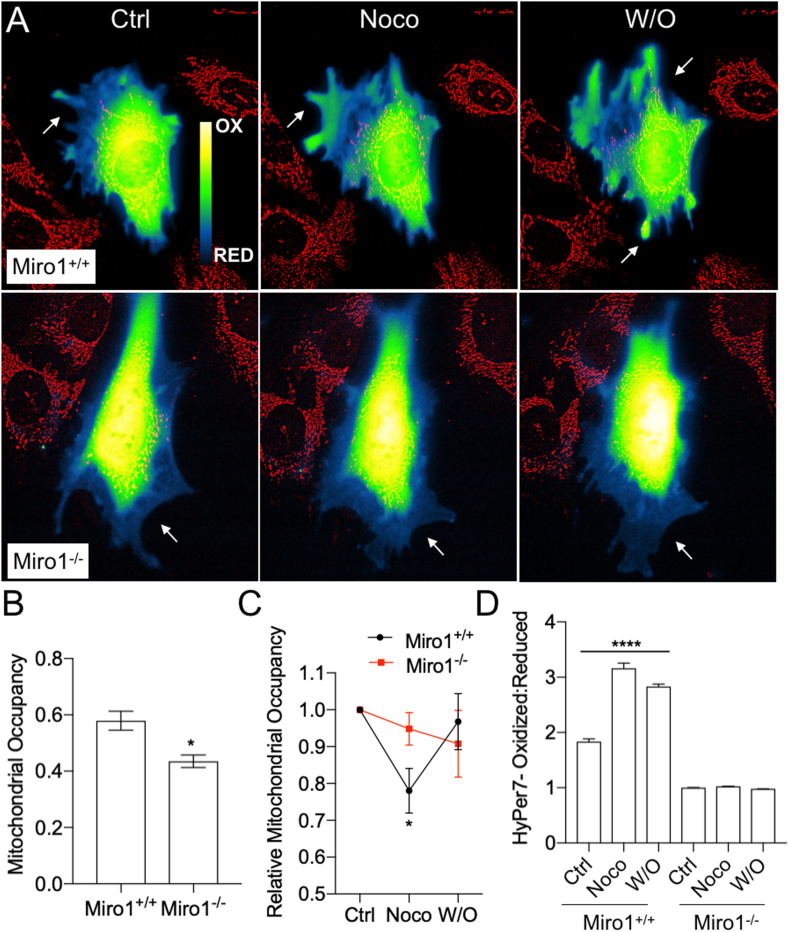
Nocodazole increases cellular H_2_O_2_ levels independent of mitochondrial occupancy but dependent on Miro1 expression. (A) Miro1^+/+^ and Miro1^−/−^ MEFs expressing HyPer7 and stained with MitoTracker Deep Red during Control (Ctrl), nocodazole (Noco) treatment and following washout (W/O) of Noco. Arrows point to peripheral areas of increased HyPer7 oxidation in Miro1^+/+^ MEFs and areas of no change in HyPer7 oxidation in Miro1^−/−^ MEFs. (B) Mitochondrial occupancy of Miro1^+/+^ and Miro1^−/−^ MEFs in control conditions (*p < 0.05; Student's *t*-test). (C) Relative mitochondrial occupancy of Miro1^+/+^ and Miro1^−/−^ MEFs following treatment (Noco) and washout (W/O) of Noco compared to control conditions (*p < 0.05; one-way ANOVA with Tukey's posttest). (D) Peripheral HyPer7 Oxidation status in Miro1^+/+^ and Miro1^−/−^ MEFs following treatment (Noco) and washout (W/O) of Noco (****p < 0.0001; one-way ANOVA with Tukey's posttest). Error bars show ± SE. (For interpretation of the references to colour in this figure legend, the reader is referred to the Web version of this article.)

We next evaluated the effects of Noco on mitochondrial distribution and HyPer7 oxidation. Noco lead to significant mitochondrial occupancy reduction in the peripheral cytoskeleton of Miro1^+/+^ MEFs (Fig. 6), similar to Miro1^−/−^ MEFs or treatment with Taxol. We did not see a significant change in peripheral mitochondrial occupancy in Miro1^−/−^ MEFs treated with Noco (Fig. 6). Interestingly, leading edge HyPer7 oxidation was significantly increased in Miro1^+/+^ MEFs following 30-min treatment with Noco, which was sustained following washout of the compound and redistribution of mitochondria into the leading edge (Fig. 6). No change in HyPer7 oxidation was observed in Miro1^−/−^ MEFs treated with Noco or following washout-out of the compound (Fig. 6). Although treatment with Noco for longer periods of time (16 h) to induce mitotic arrest also increases cellular ROS [51], increased ROS following exposure to Noco at acute timepoints, or in relationship to mitochondrial positioning, has not been described. Our data indicates Noco dependent increases in cytosolic H_2_O_2_ are dependent on Miro1 expression through a yet undetermined mechanism.

### 2.6 Focal adhesion size and abundance correlate with local H_2_O_2_ levels

**Fig. 7:**
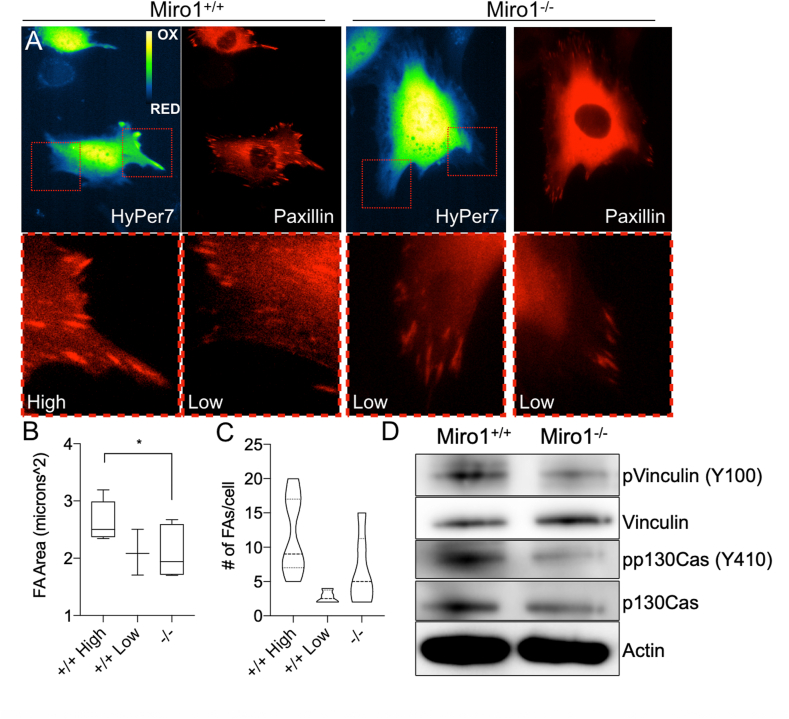
Paxillin containing focal adhesion size and abundance correlates with local H_2_O_2_ levels. (A) Miro1^+/+^ and Miro1^−/−^ MEFs expressing mCherry-Paxillin and HyPer7. Red-box inserts show mCherry-Paxillin features below at areas of high and low HyPer7 oxidation levels in Miro1^+/+^ MEFs and areas of low HyPer7 oxidation in Miro1^−/−^ MEFs. (B) Quantification of focal adhesion (FA) area (mCherry-Paxillin) at sites of high and low HyPer7 oxidation levels in Miro1^+/+^ MEFs and sites of low HyPer7 oxidation in Miro1^−/−^ MEFs (n = average from 3–5 cells/group, *p < 0.05). (C) Quantification of the number of FAs (mCherry-Paxillin) per cell at sites of high and low HyPer7 oxidation levels in Miro1^+/+^ MEFs and sitess of low HyPer7 oxidation in Miro1^−/−^ MEFs (n = average from 3–5 cells/group). (D) Western blot of reduced phospho-Vinculin (Y100) and phospho-p130Cas (Y410) phosphorylation status in Miro1^−/−^ MEFs. (For interpretation of the references to colour in this figure legend, the reader is referred to the Web version of this article.)

Focal adhesion (FA) dynamics are regulated by redox dependent mechanisms [52] and Miro1^−/−^ MEFs have perturbed FA dynamics compared to Miro1^+/+^ MEFs [24]. Additionally, recent evidence utilizing HyPer7 fused to the actin binding protein LifeAct suggests that local levels of actin oxidation correlate with persistent leading-edge protrusions [40]. Therefore, we evaluated subcellular HyPer7 oxidation status in relation to FA size and abundance in cells expressing mCherry-Paxillin plasmid DNA (Fig. 7 and Supplemental Fig. 5). mCherry-Paxillin is expressed throughout the volume of the cell and is incorporated into leading FA's through local spatial and temporal signaling events that support FA maturation [53]. We identified leading edge peripheral sites of Miro1^+/+^ MEFs with differential levels of HyPer7 oxidation (High vs low) (Fig. 7A). Alternatively, and as seen in Fig. 2, Fig. 4, the peripheral levels of HyPer7 oxidation are consistently low in Miro1^−/−^ MEFs (Fig. 7A). We visualized and quantified FA area and abundance at peripheral sites in Miro1^+/+^ MEFs with differential HyPer7 oxidation status and in Miro1^−/−^ MEFs. FA area was reduced in Miro1^+/+^ MEFs at sites of low HyPer7 oxidation compared to sites of high HyPer7 oxidation, and significantly reduced in peripheral sites of Miro1^−/−^ MEFs (Fig. 7B and Supplemental Fig. 5). The number of FAs per cell was also reduced at sites of low HyPer7 oxidation in Miro1^+/+^ MEFs and in the periphery of Miro1^−/−^ MEFs (Fig. 7C and Supplemental Fig. 5). We next evaluated the phosphorylation status of key tyrosine (Tyr) residues in vinculin and p130Cas, proteins important in governing FA dynamics in MEFs [24]. The phosphorylation status of vinculin at Tyr100 and p130Cas at Tyr410 is reduced in Miro1^−/−^ MEFs compared to Miro1^+/+^ MEFs (Fig. 7D). These data provide visual and quantitative evidence that the subcellular levels of H_2_O_2_ correlate with features required FA stability, including size, abundance and phosphorylation of key proteins.

## 3 Discussion

The goal of this study was to evaluate subcellular H_2_O_2_ levels in relationship to mitochondrial distribution in MEFs. These studies extend from our previous observations that deletion of Miro1 in MEFs restricts mitochondria to the perinuclear space and alters peripheral energy levels (ATP:ADP ratio) and that the highest ATP:ADP ratio maps to sites of highest mitochondrial density in MEFs [24]. Additionally, forced recruitment of mitochondria into leading edge pseudopodia increases local mitochondrial derived ATP levels [2]. These results are in line with biochemical evidence showing that enzymes tethered to the mitochondrial membrane are less sensitive to decreases in mitochondrial ATP output compared to soluble enzymes in the cell cytoplasm [54]. These early observations identified that ATP is not freely diffusible in cells but rather forms spikes and gradients. Therefore, a membrane-less gradient of mitochondrial outputs is present in the cell cytoplasm [55,56].

Like ATP, ROS (specifically H_2_O_2_) must be produced and act locally to activate specific redox-dependent signaling events [38,39], limit off-target cysteine oxidation and prevent excess damage to DNA, lipids and proteins [57]. Therefore, sources of cellular ROS, antioxidant systems and redox-dependent targets are compartmentalized [40,41]. For example, NADPH oxidases (NOXs) are localized to various subcellular membranes and their activity is regulated by substrate availability, post-translational modifications, chaperone proteins and second messenger concentrations [58,59]. Local activation of NOXs and subsequent increases in local ROS levels activate cell signaling pathways through direct oxidation of target cysteines and inactivation of local redox-relay protein systems (PRXs/TRXs) [59].

The flux of mitochondrial ROS is also regulated through substrate availability, oxygen availability, metabolic fluxes, genetic mutations and alterations in mitochondrial dynamics [47,60]. Our results provide an additional level of regulation to the subcellular levels of mitochondrial ROS through the intracellular positioning of mitochondria. By restricting mitochondria to the perinuclear space through the deletion of the mitochondrial adapter protein Miro1, subcellular H_2_O_2_ levels are significantly reduced in the cell periphery. The resulting consequences of decreased peripheral H_2_O_2_ correlates with altered FA features required for cell migration. We also show that disruption of the microtubule cytoskeleton, the required tracks for mitochondrial transport [61], with Taxol in Miro1^+/+^ MEFs leads to perinuclear clustering of mitochondria and reduction in peripheral H_2_O_2_ levels, partially phenocopying Miro1^−/−^ MEFs. Disruption of microtubules with Noco also induced perinuclear clustering of mitochondria in Miro1^+/+^ MEFs but lead to increased HyPer7 oxidation at sites devoid of mitochondria. Prolonged treatment with Noco leads to mitotic arrest that can also induce cell death, both of which correspond with elevated ROS [51]. We are unaware of other studies, beyond the data presented herein, that have evaluated changes in ROS following acute treatments with Noco. These results are surprising as Noco did not increase HyPer7 oxidation in cells lacking Miro1 leading us to speculate that H_2_O_2_ generation in response to Noco requires initial close association of mitochondria with additional cell factors, possibly NADPH oxidases.

Additional investigation into how mitochondrial positioning and local H_2_O_2_ production influences redox targets, redox relays and the possible regulation of NOX enzymes requires further investigation. Our biochemical corroboration that rotenone increases the oxidation of cytosolic PRX2 in Miro1^+/+^ MEFs but not in Miro1^−/−^ MEFs provides evidence for further investigation into the role of PRX redox relays dependent on subcellular mitochondrial dynamics and H_2_O_2_ production [62]. We also observed elevated nuclear H_2_O_2_ and activation of oxidant responsive DNA damage response pathways in Miro1^−/−^ MEFs at baseline and following induction of mROS with rotenone. In studies using lower concentrations of rotenone (20–30 nM) in cells with intact mitochondrial trafficking, it was found that mitochondrial H_2_O_2_ was unable to transit to the nuclear compartment [40]. Other studies, using concentrations of rotenone not disclosed, found that mROS could transmit from the IMS to other cellular compartments [63], possibly indicating a threshold of ROS escape from the mitochondria may exist. This hypothesis is in line with evidence that perinuclear increases in mitochondrial density and mROS can alter transcriptional responses through direct oxidation of promoter sites [64]. Our data that rotenone increases HyPer7 oxidation in the nucleus of Miro1^−/−^ MEFs and increases phosphorylation of the DNA damage response protein H2AX provide further precedence for investigating transcriptional responses dependent on subcellular mitochondrial positioning and mROS as mitochondrial presentations and the production/metabolism of mitochondrial H_2_O_2_ varies across cell types during normal physiology and disease states [60,65].

H_2_O_2_ gradients have been shown to correlate with changes in cell migration phenotypes. ROS and in particular H_2_O_2_, act on numerous signaling pathways controlling cell migration including receptor activation, kinase and phosphatase activity, FA dynamics, membrane reorganization and transcription factor activation [52]. FAK phosphorylation, FA formation and cell spreading are all attenuated by inhibition of redox signaling [66].

Mitochondrial and NOX-dependent sources of ROS have both been implicated in regulation of these processes, but due to the intimate crosstalk between mitochondria and NOX enzymes [67], deciphering the precise contribution from each source has been challenging. Our data provides new evidence that differences in H_2_O_2_ levels at subcellular sites correlates with changes in FA size and abundance. The presence of mitochondria is important in FA dynamics as FA dynamics are perturbed in Miro1^−/−^ MEFs [24] and key phosphorylation events in vinculin and p130Cas are significantly reduced in Miro1^−/−^ MEFs (Fig. 7). How mitochondrial abundance and redox-dependent signaling regulates cytoskeleton rearrangement and FA dynamics is still not clear. FA proteins are ubiquitously expressed in the cytoplasm and are post-translationally modified, associate with binding partners and are dynamically regulated in precise space and time to support local FA maturation [68]. Therefore, it is essential that the redox control over this process be tuned to the correct levels of ROS required. The detailed assessment of the redox status of individual mitochondria and subcellular H_2_0_2_ levels, in space and time during this process, will be required to comprehensively understand mROS contribution to the dynamic process of FA maturation and cell migration. Although the ROS source was not investigated, oxidation of key cysteine residues in the actin binding protein cofilin supports directional migration of breast cancer cells [43]. Additionally, the oxidation of two cysteine residues in actin by NOX4 is critical for vinculin binding and FA maturation during cell spreading in endothelial cells [69]. The van der Vliet group has also provided evidence that actin and Src oxidation via DUOX1 supports epithelial cell migration [70]. Conceivably, these oxidation events can occur at various subcellular sites but are support cell attachment and migration when confined to specific subcellular sites. Our data presented here, and the literature supporting NOX-dependent redox modifications in support of cell migration, indicate a need to critically evaluate mitochondrial and NOX crosstalk in supporting dynamic redox-regulated events in subcellular space and time.

In conclusion, this study provides evidence that the subcellular distribution of mitochondria via Miro1-mediated trafficking on microtubule tracks supports subcellular H2O2 gradients. These findings further support the requirement for precise localization and activation of ROS generating systems in dictating subcellular ROS levels.

## 4 Methods

### 4.1 Cell lines and cell culture

*Miro1* ^*+/+*^ and *Miro1*^*−/−*^ mouse embryonic fibroblast (MEF) cell lines previously described in Ref. [27] were obtained from the lab of Dr. Janet Shaw at the University of Utah. The Myc-Miro1 cell line was created as described below. Cells were maintained in antibiotic-free DMEM media (Gibco) supplemented with 4.5 g/L d-glucose, 10% fetal bovine serum (FBS, Corning), l-Glutamine, and 75.6 μM Beta-Mercaptoethanol (Sigma) at 37°C in a humified incubator containing 5% CO2.

### 4.2 Generation of stable Myc-Miro1 expressing cell line

Miro1^−/−^ MEFS were transfected with Myc-Miro1 plasmid DNA (A kind gift from Dr. Janet Shaw) using a Neon electroporation system following the manufacturer's protocol for transfecting MEFs (Invitrogen 10-μl kit). Briefly, 400,000 cells were centrifuged at 2500×*g* for 4 min, washed once with 1X PBS and centrifuged again in 1X PBS for additional 4 min at 2500×*g*. Cell pellets were resuspended in 10 μl R buffer containing 1.5 μg of Myc-Miro1 plasmid. Myc-Miro1 plasmid was transfected into cells using electroporation with a 1350 V pulse voltage, 30 ms pulse width and 1 pulse number settings. Transfected cells were then plated in selection media containing 3–5 mg/ml Hygromycin B in which they were propagated in for 2–3 months. For experiments, Myc-Miro1 MEFs were switched back to antibiotic free media and their Miro1 expression levels was assessed by qPCR monthly using Miro1 primers spanning exons 2–4.

### 4.3 Drug treatment of cells

In experiments that involved exposing cells to drug compounds, cells were treated with 1 μM Rotenone (Sigma), 0.5 μM Taxol (Sigma), 0.5 μM nocodazole (Sigma) or 10 mU/ml glucose oxidase (Sigma) for indicated periods of time in cell culture media or imaging media.

### 4.4 Western blotting

Following drug treatment, cells were incubated with 100 mM methyl methanethiol sulfonate (MMTS) diluted in 1XPBS on ice as previously described [71] for 20 min to prevent the oxidation of free thiol groups. MMTS was removed, cells were washed twice with 1X PBS and lysed with 1% Triton x-100 lysis buffer supplemented with 1 mini-protease tablet (Thermo Scientific). To clear cellular debris, lysates were centrifuged at 14,000 rpm for 10 min and protein concentrations were determined by a Bradford assay. Protein lysates (15 μg protein/well) were resolved under reducing or non-reducing conditions (reducing agent was omitted from the sample buffer) on 4–12% Tris SDS-PAGE gradient gels (Invitrogen) and transferred to polyvinylidene fluoride (PVDF) membranes (GE Health Care Life Science) for immunoblotting. Membranes were blocked with 5% bovine serum albumin (BSA) in 1X Tris-Buffered saline (TBS) containing 0.2% tween (TBST) at 4C overnight and immunoblotted with PRX2 (1:2000, R&D Systems, Catalog # AF3489), PRX3 (1:1000, AbFrontier Catalog # LF-PA0255), pVinculin (1:1000, ThermoFisher Catalog # 44–10746), Vinculin (1:2000, ThermoFisher, Catalog # 14–9777–80), pp130Cas (1:000, Cell Signaling Technologies, Catalog # 4011), p130Cas (1:1000, Cell Signaling Technologies, Catalog # 13846) or GAPDH (1:3000, Invitrogen, Catalog# ma5 15788) in blocking buffer overnight at 4C. Membranes were washed the following day 6–8 times for 30 min with TBST and incubated with horseradish peroxidase (HRP)- conjugated goat (Invitrogen), mouse (GE Health Care), or rabbit (GE Health Care) secondary antibodies for 1 h. Following secondary antibody incubation, membranes were washed with TBST for 1 h and enhanced chemiluminescent substrate (Thermo Scientific) was used to detect HRP-conjugated secondary antibodies. Images were collected on a GE imaging station and analyzed using ImageJ and GraphPad Prism software.

### 4.5 HyPer7 plasmid transfection and imaging

HyPer7 plasmid originally obtained as a bacterial stock from Addgene (Plasmid #136466) and previously described in Refs. [40] was grown in antibiotic selection media. HyPer7plasmid DNA was isolated from bacterial cultures using a Midi-prep plasmid preparation kit (Invitrogen). Plasmid DNA was sequenced to confirm the presence of HyPer7. Cells were transfected with HyPer7 plasmid using a Neon electroporation system following the manufacturer's protocol for transfecting MEFs (Invitrogen 10-μl kit). Briefly, 400,000 cells were centrifuged at 2500×*g* for 4 min, washed once with 1X PBS and centrifuged again in 1X PBS for additional 4 min at 2500×*g*. Cell pellets were resuspended in 10 μl R buffer (Invitrogen) containing 1.5 μg of HyPer7 plasmid. HyPer7 was transfected into cells using electroporation with a 1350 V pulse voltage, 30 ms pulse width and 1 pulse number settings. Transfected cells were then plated on top of glass cover slips in a 6 well plate containing antibiotic-free DMEM cell culture media.

Cells (5–10 per experiment) were imaged 24 h after transfection. In experiments visualizing mitochondria, prior to imaging, cells were stained with 0.25 μM MitoTracker Deep Red (Invitrogen) for 15 min in complete media. MitoTracker Deep Red was removed, and cells were allowed to equilibrate for 60 min in fresh media (this greatly increases the signal to noise ratio). Cover slips were placed in a cover slip holder and maintained in CO_2_-independent imaging medium containing 134 mM NaCl, 5.4 mM KCl, 1.0 mM MgSO4, 1.8 mM CaCl2, 20 mM HEPES, and 5 mM d-glucose (pH 7.4). A stage warmer was used to maintain cells at 37°C while cells were imaged on an Eclipse TE- 2000E inverted microscope (Nikon) equipped with a 40×/1.3 numerical aperture (NA) Plan Fluor oil-immersion objective. Emission was collected sequentially at 510/40-nm following excitation with the 395-nm LED and the 470-nm LED by a Spectra X LED light engine (Lumencor, Beaverton, OR). Images of mitochondria were collected following excitation at 555-nm with emission at 624/40-nm. Images were collected by a Clara charge-coupled device camera (Andor, Concord, MA) as previously described [2]. Images were acquired every 1 min with 700 ms exposure time and analyzed using ImageJ software. See Supplemental Fig. 3 for workflow. Kymographs were generated using the reslice function in ImageJ.

### 4.6 Analysis of HyPer7 oxidation in relation to mitochondria

To determine the ratio of HyPer7 oxidation (oxidized: reduced) individual channels representing the reduced version of the probe (Ex.395/Em510) and the oxidized version (Ex.470/Em510) were combined using the Image Calculator command in ImageJ (Ex.470 ÷ Ex. 35). Raw values of the oxidized: reduced ratio or values derived from normalizing to the lowest signal were used for subsequent analysis. Mitochondria images were uniformly adjusted for brightness/contrast, and a “top-hat” filter was applied to isolate bright mitochondria from background [2]. Filtered images were used to create binary masks and regions of interest associated with individual mitochondria were applied to the HyPer7 combined image to determine HyPer7 oxidation status at areas associated and not associated with mitochondria. All images were uniformly adjusted for brightness/contrast and therefore the HyPer7 ratio presented is comparable across cell types. See Supplemental Fig. 3 for workflow. To evaluate the relationship between HyPer7 oxidation and mitochondrial abundance in leading edge protrusions over time, kymographs were generated using reslice function in ImageJ and mitochondrial fluorescence intensity and HyPer7 ratio were determined from leading edge ROIs using the plot profile function in ImageJ.

## 5 mCherry-Paxillin imaging and analysis

Cells were transfected with mCherry-Paxillin (Addgene #50526) in combination with HyPer7 as described above. Cells (5–10 per experiment) were imaged 24 h after transfection as described above. Focal adhesion size and abundance was quantified following particle analysis (1.5 μm^2–15 μm^2) of mCherry-Paxillin images subjected to equal thresholding.

### 5.1 Immunofluorescence of phospho-H2AX staining

Cells were grown on glass coverslips in a 6-well plate at 75, 000 cells per well confluency. The following day, cells were treated with different rotenone concentration (1–10 μM) for 2 h. Cells were then fixed with 4% Paraformaldehyde for 10 min, washed once with 1 X PBS, incubated in 0.25% Permeabilization buffer containing Triton X-100 (Fisher Bioreagents), washed once with 1 X PBS and then incubated in 1.5% BSA in 1X PBS for 24–72 h. The slides were stained with gamma-pH2AX Antibody (1:500, EMD Millipore Corp, Catalog # 05–656), washed, and then incubated with an Alexa-647-conjugated anti-mouse IgG (1:1000, Invitrogen, REF# A31571) and DAPI to visualize gamma-pH2AX and the nucleus. Images were acquired using a Nikon A1R confocal microscope. Briefly, multiple z-planes at 0.5 μm intervals were collected to visualize the entire volume of the cell. Images were compressed into a maximum intensity projection (MIP) prior to analysis. pH2AX intensity was calculated for each cell by drawing a mask around the nucleus using the DAPI signal and determining the mean fluorescence intensity for H2AX in each nucleus.

Measurement of extracellular H_2_O_2_ with Amplex Red.

Miro1^+/+^ and Miro1^−/−^ MEFs were plated into a 96-well plate and allowed to adhere overnight. The following day cells were incubated with Amplex Red (Invitrogen) following the manufactures instructions. H_2_O_2_ produced from cells was monitored by following the fluorescence of resorufin using a BioTek Synergy plate reader set to collect Emission at 585-nm following excitation at 565-nm for 60 min. Experiments were conducted at 37C. At the completion of the experiment cells were incubated with Hoechst dye and total cellular content was determined. Wells containing no Amplex Red provided no signal for either cell line (not shown). Amplex Red signal was normalized to total cellular content.

## Declaration of competing interest

Dr. Cunniff serves as a consultant for RS Oncology and retains equity in the company. RS Oncology supports additional efforts underway in the Cunniff Lab not directly related to research presented within.

## Appendix A Supplementary data

figs1 figs1

figs2 figs2

figs3 figs3

figs4 figs4

figs5 figs5
