# Supplementary figures and images for: Miro1-mediated mitochondrial positioning supports subcellular redox status
Source: Redox Biol. 2020 Nov 29;38:101818. doi: 10.1016/j.redox.2020.101818 (PMC7753203; doi:10.1016/j.redox.2020.101818)

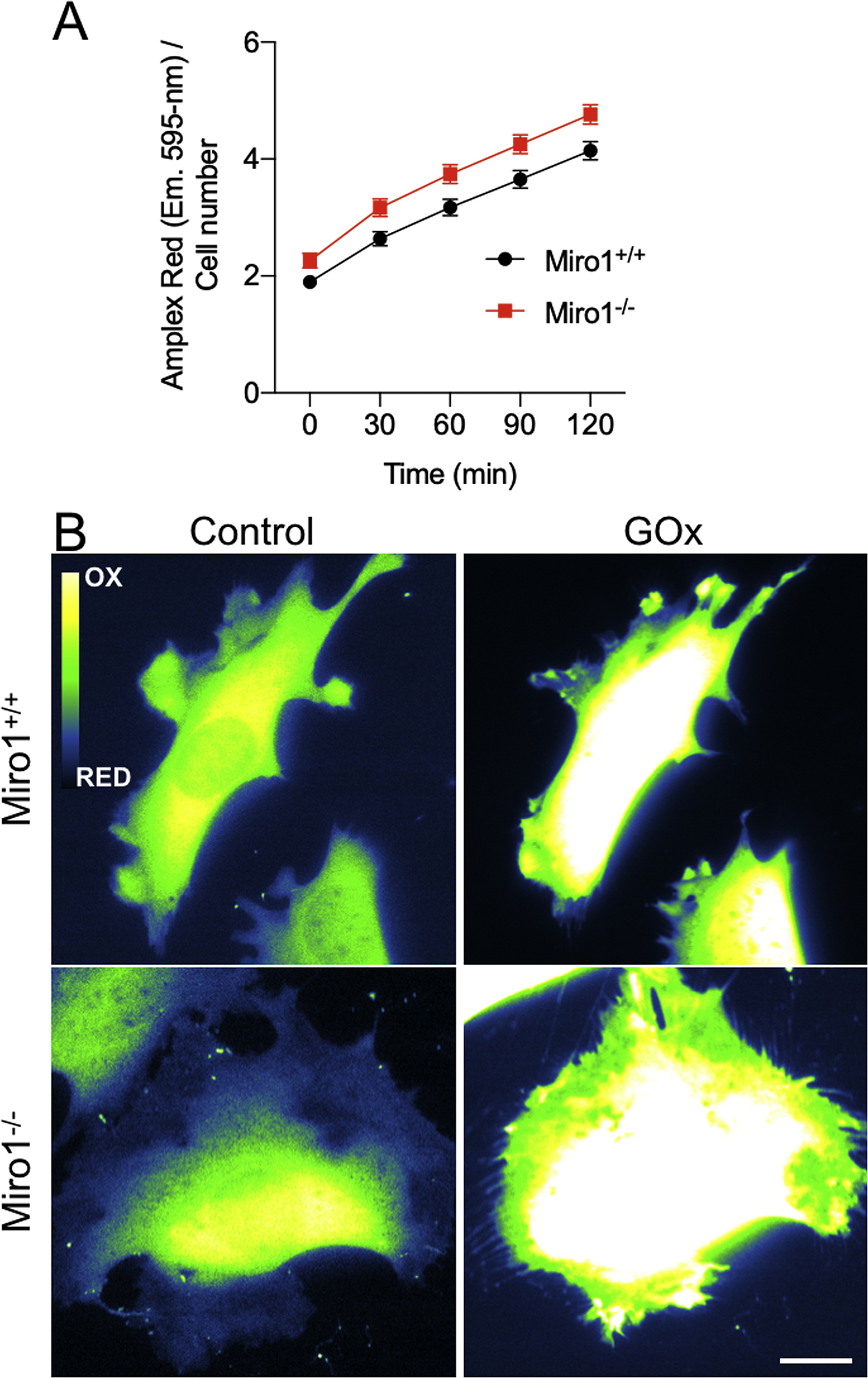

Supplement: figs1 [file mmcfigs1.jpg]

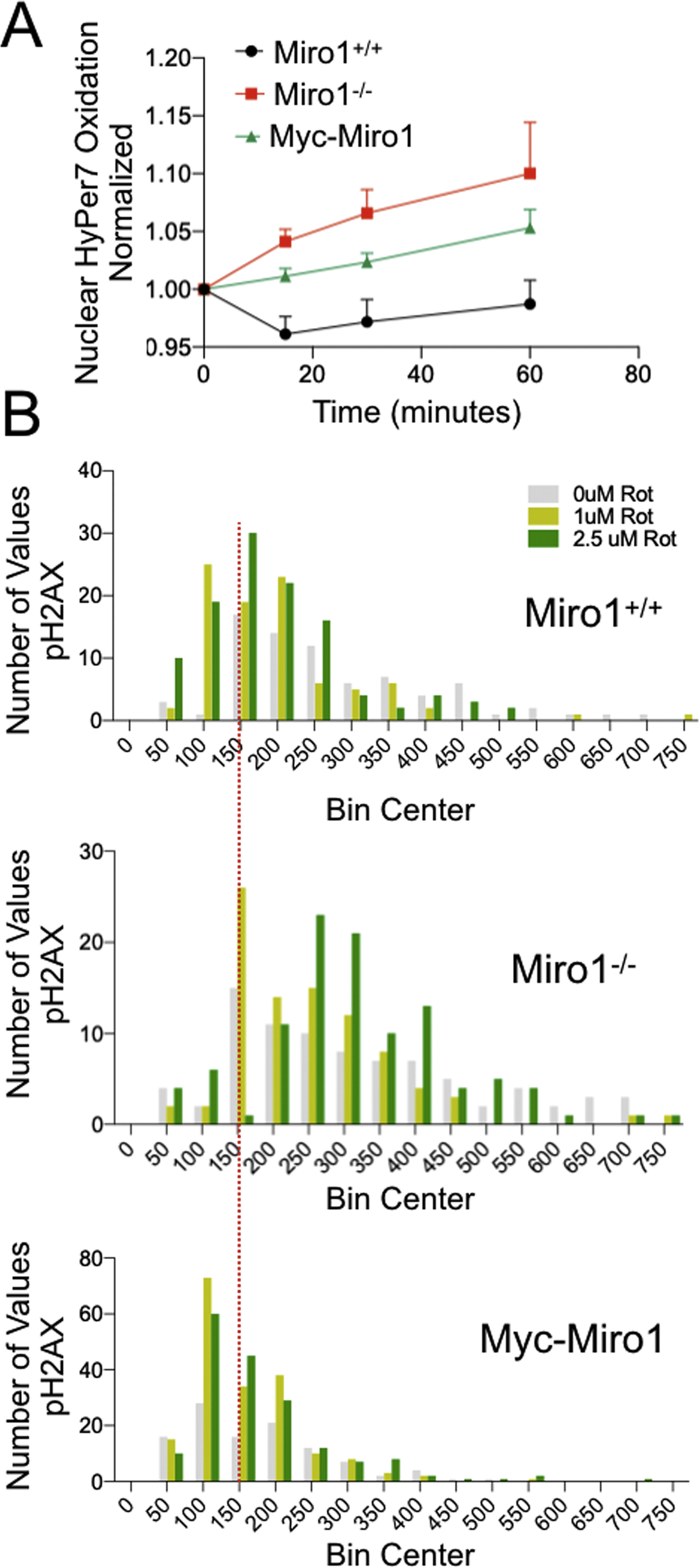

Supplement: figs2 [file mmcfigs2.jpg]

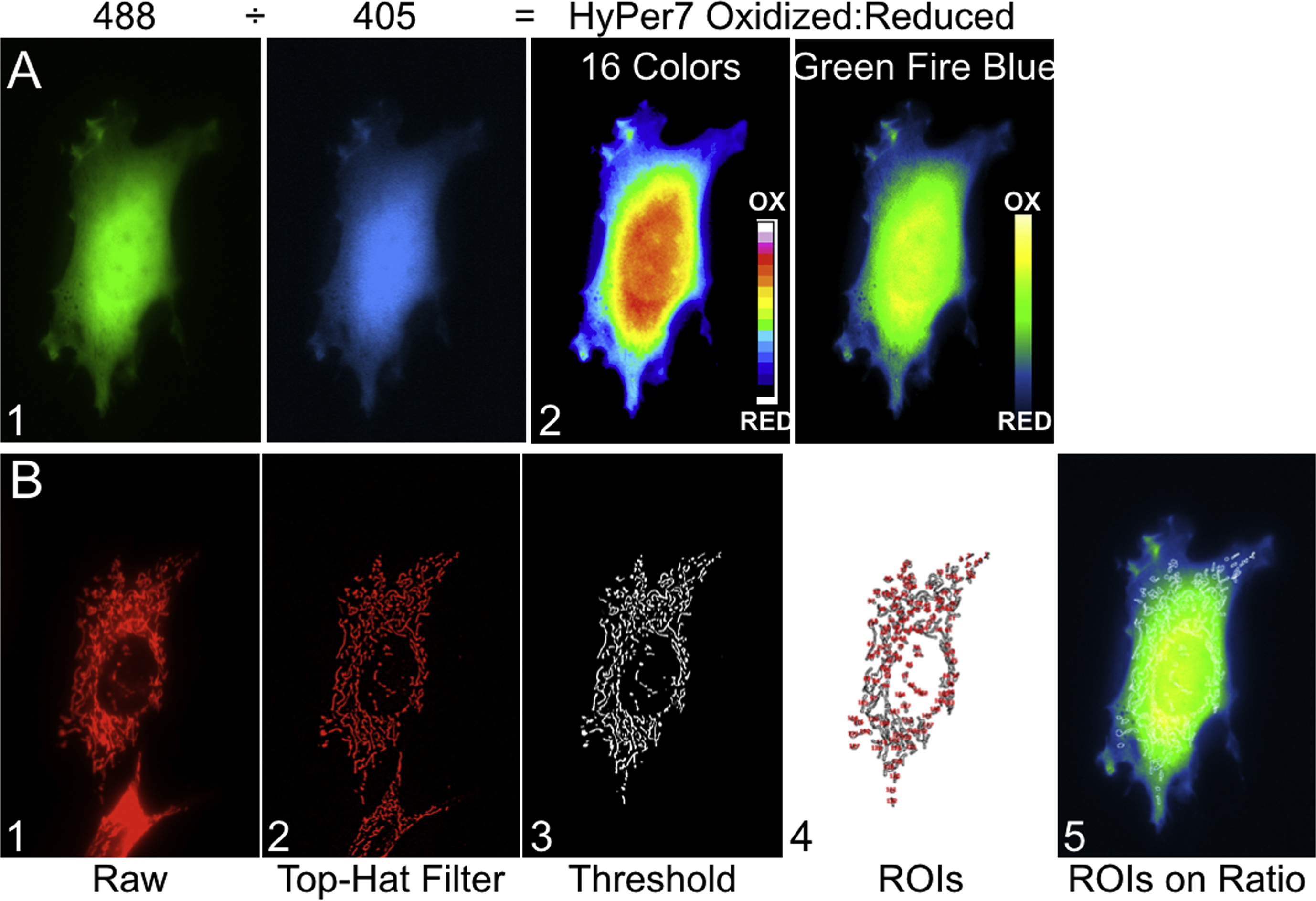

Supplement: figs3 [file mmcfigs3.jpg]

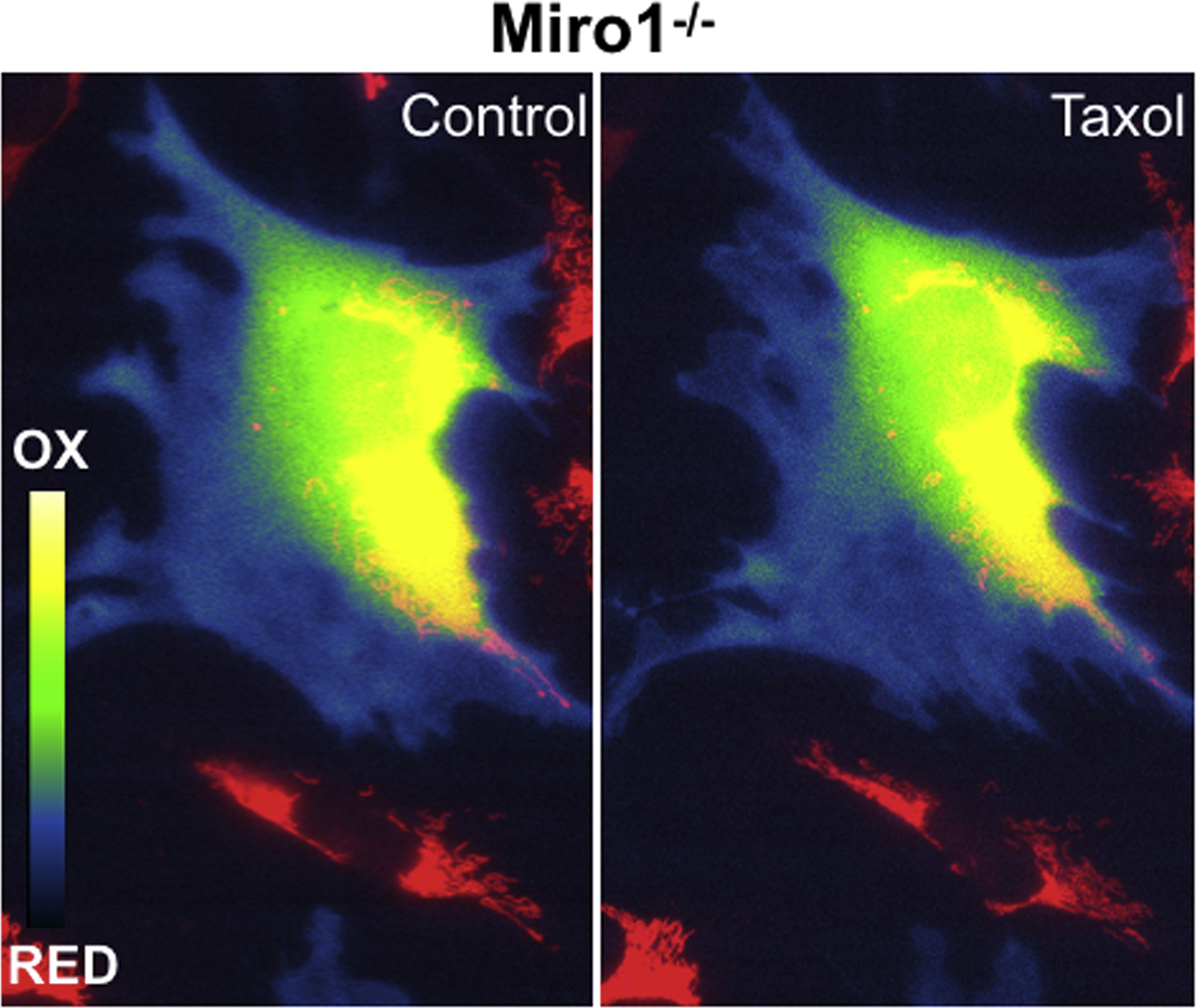

Supplement: figs4 [file mmcfigs4.jpg]

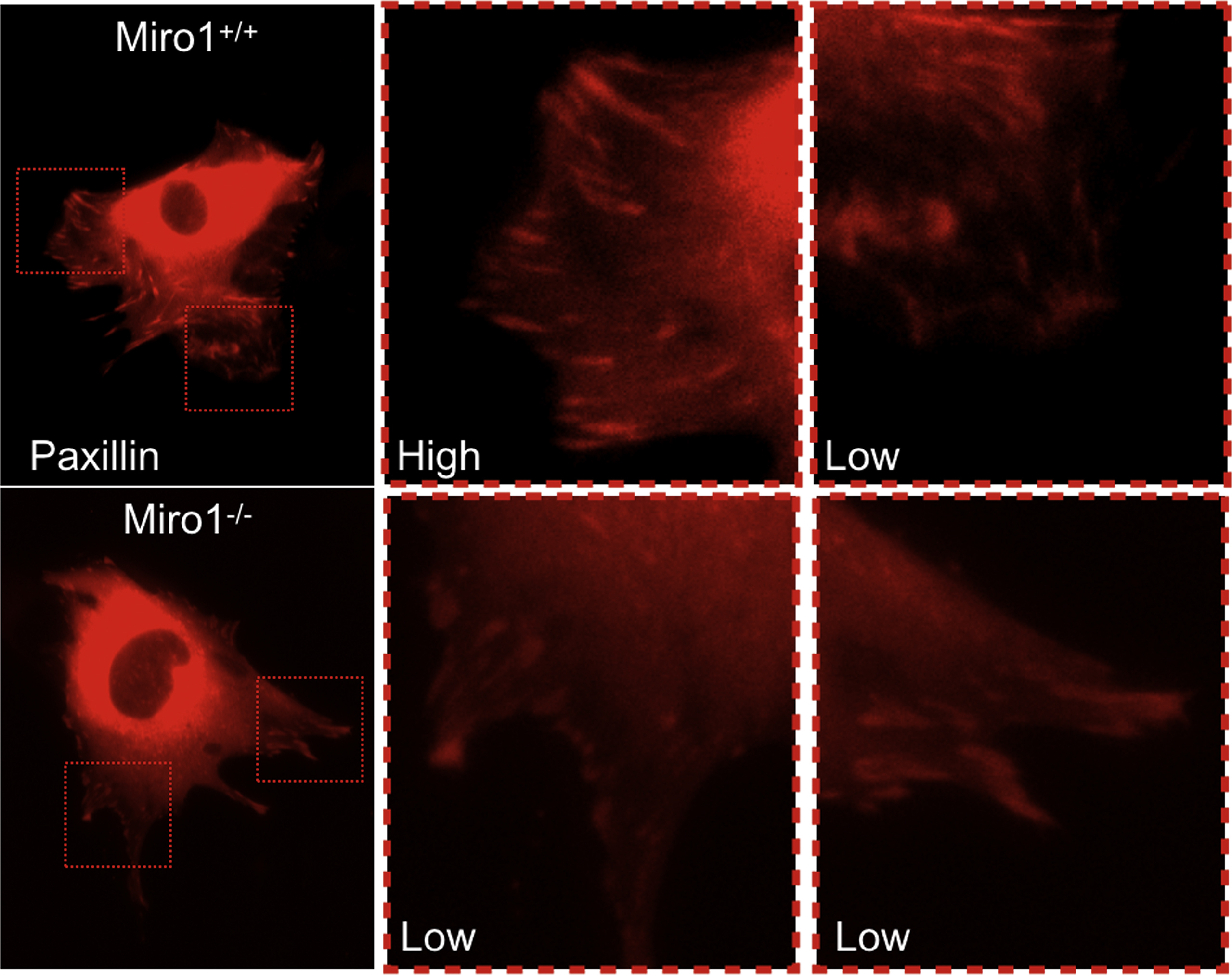

Supplement: figs5 [file mmcfigs5.jpg]
